# Relationship Between Chronotype, Sleep Disorders, and Temperament in Adolescents with Attention Deficit Hyperactivity Disorder

**DOI:** 10.1192/j.eurpsy.2025.1116

**Published:** 2025-08-26

**Authors:** I. Cihanyurdu Erdem, A. T. Bahadır

**Affiliations:** 1 Child and Adolescent Psychiatry, Marmara University Faculty of Medicine, Istanbul, Türkiye

## Abstract

**Introduction:**

Studies focusing on the relationship between chronotype, sleep disorders and temperament traits in Attention-Deficit/Hyperactivity Disorder (ADHD) are limited, particularly in the context of adolescents. In adolescents aged 11 to 18 diagnosed with ADHD, we hypothesize that chronotype, sleep problems, and temperament traits may be dynamically interrelated across a broad spectrum. Conducting in-depth studies to examine these relationships could enhance our understanding of ADHD treatment and management strategies, potentially leading to more effective interventions.

**Objectives:**

We aimed to explore the relationship between chronotype characteristics, sleep disturbances, and temperament traits among adolescents diagnosed with ADHD and to compare them with healthy controls.

**Methods:**

Participants included 102 adolescents aged 11 to 18 years with an ADHD diagnosis from the Child and Adolescent Psychiatry outpatient clinic at Marmara University Pendik Training and Research Hospital, along with 101 age-matched controls from a secondary and a high school. Clinical assessments were conducted using the K-SADS-PL. Parents completed the Sleep Disturbance Scale for Children and Children’s Chronotype Questionnaire while adolescents completed the Junior Temperament and Character Inventory Revised. These assesments evaluated chronotype characteristics, sleep disturbances, and temperament traits.

**Results:**

Adolescents with ADHD demonstrated significantly higher rates of sleep disturbances and delayed chronotype compared to the controls. Additionally, adolescents with ADHD exhibited significantly higher levels of novelty seeking and harm avoidance scores, while the control group exhibited higher levels of persistence and self-directedness compared to the ADHD group. In the ADHD group, chronotype total score correlated negatively with self-directedness (r=-0.201, p=0.042) and reward dependence (r=-0.199, p=0.045). Novelty seeking was pronounced in those with an evening chronotype and positively correlated with severe sleep disturbances, including difficulties initiating and maintaining sleep (r=0.210, p=0.034). Logistic regression analyses indicated that novelty seeking significantly predicted evening chronotype, while linear regression revealed that both novelty seeking and chronotype scores significantly predicted overall sleep disturbance severity. (R2=0.112, p=0.006).

**Conclusions:**

This study represents one of the initial investigations emphasizing the intricate relationships between chronotype characteristics, sleep disturbances, and temperament traits in adolescents diagnosed with ADHD. More profoundly underscoring these relationships and their clinical significance could be crucial for targeted interventions to improve ADHD management.

**Disclosure of Interest:**

None Declared

